# New therapeutic approach for tricuspid regurgitation: Transcatheter tricuspid valve replacement or repair

**DOI:** 10.3389/fcvm.2023.1080101

**Published:** 2023-02-23

**Authors:** David I. Blusztein, Rebecca T. Hahn

**Affiliations:** Division of Cardiology, Columbia University Irving Medical Center, New York, NY, United States

**Keywords:** tricuspid regurgitation, transcatheter, French size, superior vena cava, inferior vena cava, treatment

## Abstract

The tricuspid valve is a complex structure with normal function dependent on the leaflet morphology, right atrial and annular dynamics, and right ventricular and chordal support. Thus, the pathophysiology of tricuspid regurgitation (TR) is equally complex and current medical and surgical management options are limited. Transcatheter devices are currently being investigated as possible treatment options with lower morbidity and mortality than open surgical procedures. These devices can be divided by their implant location/mechanism of action: leaflet approximation devices, annuloplasty devices, orthotopic valve implants, and heterotopic valve implants. The current review will discuss each class of transcatheter device therapy, and further delve into the current understanding of who and when to treat. Finally, we will include a brief discussion of the future of device and surgical therapy trials for TR and the remaining questions to answer about this complex disease process.

## Introduction

Severe tricuspid regurgitation (TR) is associated with significant morbidity and reduced life expectancy (1, 2) that historically has been left untreated. Associated with atrial fibrillation, increased bleeding risk and multiorgan dysfunction, TR has become a focus of innovation to allow for reversibility of these consequences seen in chronic right heart failure (3). When symptoms of dyspnea, fatigue and edema are unresponsive to medical therapy, patients pursue definitive valve intervention. Surgical tricuspid valve (TV) repair and replacement have classically been the primary mode of intervention with unclear benefit compared to medical therapy and significant peri-procedural risk (4). A high in-hospital mortality rate of 9–10% for isolated tricuspid valve surgery (5, 6) has been attributed to delays in timing of intervention in highly symptomatic patients with multiple co-morbidities and right heart dysfunction.

Transcatheter tricuspid valve interventions (TTVI) have the potential for reducing the acute procedural and in-hospital adverse outcomes associated with cardiac surgery. Given the large underserved patient population and early reports of safe and effective outcomes after TTVI, there has been rapid development of devices to treat TR. Whether TTVI will improve survival compared to medical therapy may be answered in the randomized control trials currently enrolling. This review aims to outline the current understanding of TR etiology and its associated cardiac morphology, options for TTVI including device-specific characteristics and the required pre-procedural evaluation, as well as considerations for device choice. Finally, available TTVI outcomes data may offer a glimpse into what we can expect from the current pivotal trials.

### Tricuspid regurgitation etiology and morphology

Historically the etiology of TR has been simplistically divided into primary (i.e., leaflet abnormality) and secondary (i.e., intrinsically normal leaflets with malcoaptation) however our current etiologic classification now reflects a more comprehensive understanding of the morphologic differences not only of the leaflets, but of the right atrial (RA) and right ventricular (RV) anatomy (7, 8). Primary TR continues to include all disease states with altered tricuspid leaflet anatomy and function: congenital heart disease, infiltrative or inflammatory disease (i.e., carcinoid or rheumatic), endocarditis and trauma. Cardiac implantable electronic device (CIED) related TR is no longer considered primary, since the presence of the device and the mechanism of TR is not an isolated leaflet issue. There are 2 types of patients with CIEDs and TR: those in whom the device is directly responsible for the TR due to leaflet or subvalvular interference (i.e., impingement, entanglement, adhesions, or perforation) and those in whom the device is incidental with other etiologies of TR. Secondary TR, whereby leaflet structure is preserved, is now divided into atrial and ventricular subcategories. Atrial secondary TR is a consequence of RA enlargement and dysfunction causing dominant TV annular dilatation, commonly due to atrial fibrillation/flutter, heart failure with preserved ejection fraction and age. This pathology does not classically result in leaflet restriction or tethering and RV abnormalities may be absent. Ventricular secondary TR is a consequence of RV enlargement and/or dysfunction causing annular dilatation with TV leaflet tethering and restriction in systole secondary to left-sided ventricular or valve disease, pulmonary hypertension (PHT) and RV dysfunction of any cause including myocardial diseases, ischemic heart disease and chronic RV pacing. Much like secondary mitral regurgitation, secondary TR begets more TR, culminating in divergence of the interventricular septum towards the left ventricle (LV), restricting its filling and worsening RV afterload conditions due to increased LV diastolic and pulmonary artery (PA) pressures.

**Figure 1:**
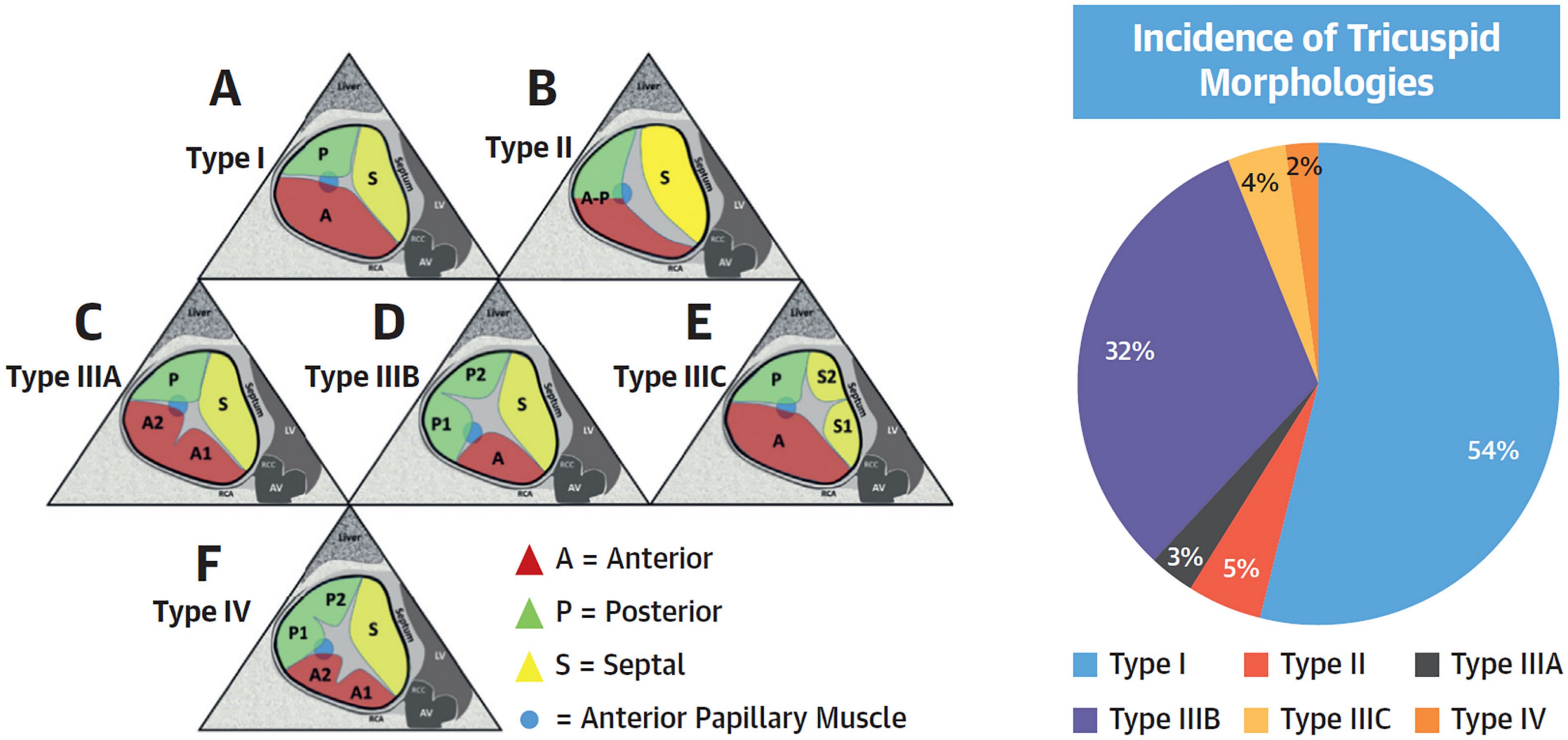
Tricuspid Valve Nomenclature Classification Scheme. **(Left)** A proposed tricuspid valve nomenclature classification scheme is shown. The anterior papillary muscle is indicated as a **blue circle** and defines the separation of the anterior from the posterior leaflets. **(A)** Type I: 3-leaflet configuration. **(B)** Type II: 2-leaflet configuration. **(C–E)** Type III: 4-leaflet configurations. **(F)** Type IV: 5-leaflet configuration. **(Right)** Incidence of each morphology in the present study of 579 patients. Adapted version from Dr. Hahn with permission.

Understanding the mechanism of TR is important when considering the best transcatheter treatment options given the various anchoring mechanisms and methods of action for each device. For example, transcatheter edge-to-edge repair (TEER) or annular repair devices will likely not eliminate CIED-related TR whereas replacement devices may be effective. TR related to significant leaflet tethering seen with ventricular secondary TR may not respond to an isolated annular device although TEER devices can result in significant TR reduction. TV leaflet variability (9) has recently been recognized as a potential predictor of TEER procedural failure (10). Thus, consideration of TR etiology and morphology through detailed pre-procedural imaging and evaluation of physiological conditions is paramount for appropriate patient selection, procedural success, and durable TR reduction (Figure 1).

### Imaging of the tricuspid valve

Thorough pre-procedural imaging with cardiac computed tomography (CCT) and both transthoracic (TTE) and transesophageal echocardiography (TEE) allow for more specific pre-procedural planning with strengths and weaknesses of each modality. Importantly, TR varies not only with respiratory cycle and rhythm, but also with volume status. Thus irrespective of the imaging method, the assessment of disease severity should be performed under stable, optimal guideline directed medical therapy. New guidelines describe the utility of pre-procedural echocardiography for determining the location and size of the regurgitant orifice (and thus TR severity), evaluating leaflet and ventricular morphology (and thus TR etiology), assessing RV and left ventricular size and function, and determining the severity of concomitant valvular disease or other cardiac abnormalities (11). Due to its complex structure, 3D echocardiography is superior to conventional 2D evaluation in comprehensively evaluating TR etiology and severity as well as quantitating annulus size and right heart chamber function. (12, 13) Given the anterior position of the right heart, TTE imaging often results in adequate 3D volume acquisitions however 3D TEE evaluation requires an experienced operator in order to overcome imaging difficulties relating to the far-field and off-axis position of the TV relative to the esophagus, the non-circular nature of the tricuspid annulus and that the leaflets are thin and often oblique to the ultrasonography beam. Where 2D TTE annular measurements may underestimate size, 3D TEE measurements are comparable to cardiac magnetic resonance imaging (CMR), CCT, and surgical measurements (14–16). This enhanced detail becomes important in TTVI evaluation with regards to device type, size, and feasibility.

Because patients present late in the disease process, five-grade TR severity scale (17) expands the “severe” grade into 2 additional grades: mild (1+), moderate (2+), severe (3+), massive (4+), and torrential (5+). This grading scheme accounts for the large cohort in current trials that markedly exceed the historical severe TR echocardiographic criteria, findings that have been associated with adverse RV remodeling and mortality (18, 19). The grading scheme is based on evaluating the average vena contracta width (in two orthogonal views to account for its crescent-shaped orifice), effective regurgitant orifice area (EROA) by proximal isovelocity surface area (PISA), 3D vena contracta area or quantitative EROA. The latter measurement quantifies transtricuspid diastolic stroke volume by either measuring 2D orthogonal annular measurements and applying an elliptical area formula, or by direct planimetry of the annular area on 3D multi-planar reconstruction. Recent studies have shown that PISA measurements of EROA underestimate 3D vena contracta area and quantitative Doppler EROA by up to 40% (20–22). Pre-procedure TEE may also identify potential procedural challenges, including difficulty to obtain detailed imaging, and may factor into selection of transcatheter therapy.

**Figure 2:**
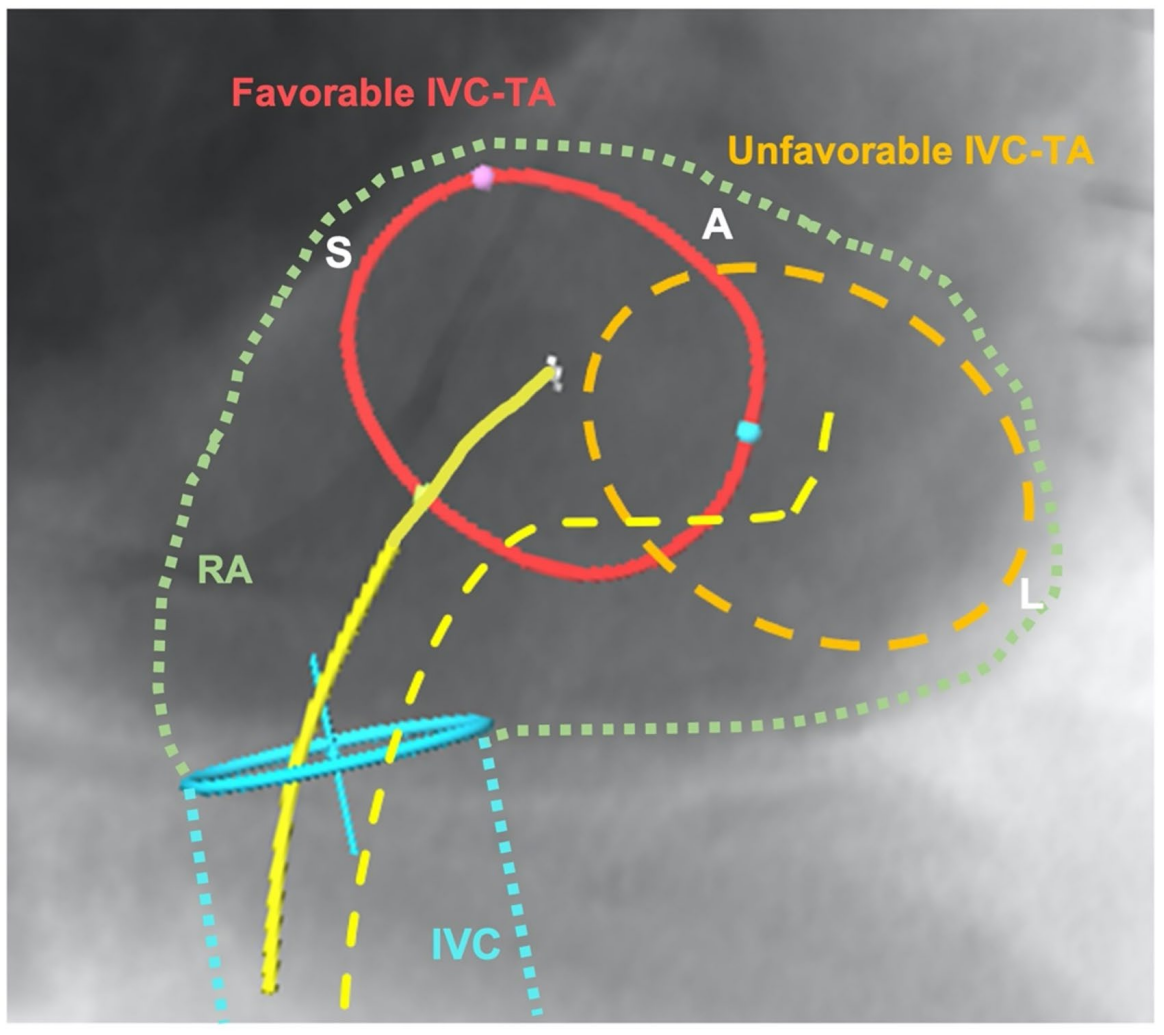
Illustration of favorable and unfavorable relationship between inferior vena cava and tricuspid annulus (IVC-TA). This figure demonstrates a short-axis view of the tricuspid annulus and the inferior vena cava course (blue) and entry into the right atrium (green) using CT-fluoroscopic fusion imaging. The favorable anatomy (red) shows no major offset compared to the unfavorable IVC-TA, where the annulus is completely offset laterally from the IVC origin. The yellow lines depict the secondary flexion required to achieve coaxiality, with the dotted line requiring severe secondary flexion, a movement that will impact primary movements and ability to be coaxial with tricuspid valve. IVC, inferior vena; TA, tricuspid annulus; RA, right atrium; S, septal; A, anterior; L, lateral.

CCT is essential for the evaluation of annular size, CIED lead course, right coronary artery proximity and sub-valvular structures (e.g., papillary muscles). CCT is also essential for the determination of appropriate access: venous access site dimensions and tortuosity as well as caval anatomy and approach to the tricuspid valve. A three dimensional (3D) understanding of vena caval course and angulation is important for guide-catheter positioning to allow for coaxial approach. The inferior vena caval to tricuspid annulus relationship (Figure 2) is highly variable and may be a significant determinant of technical success. The inferior vena caval height above the tricuspid annulus and its offset to the annulus determine the degree of primary and secondary flexion, respectively, that will be required to achieve coaxiality.

### Physiologic considerations

Beyond anatomical considerations, a comprehensive understanding of physiological loading conditions is important in patient selection and outlook with valve intervention. This encompasses the conditions faced by the right ventricle and may help better understand the contribution of left-sided ventricular or valvular disease and PHT to best evaluate the likely benefit and safety of TV intervention. A multi-modal evaluation that utilizes non-invasive and invasive investigations to best quantify RV size and function, PA pressures, left heart hemodynamics and pulmonary vascular resistance should be performed, ideally in a euvolemic state.

The RV has long been thought to be sensitive to afterload. Recent studies suggest that a PA pressure of ≥36 mm Hg, left atrial dilatation, age and atrial fibrillation are strong predictors of TR progression (23). Chronic significant TR subsequently imposes a volume overload on the RA and RV and a change in RV pressure which, in the restricted pericardial space, may have an impact on LV function. Over time, there is maladaptive remodeling of the RV with consequent reduction in RV function. A reduction of TR can potentially have two effects: it increases RV afterload, which could worsen RV systolic function, but also reduces RV volume potentially improving LV filling conditions. Using invasive pressure-volume analysis during tricuspid TEER, two studies confirmed that following a reduction in TR, there were larger LV end-diastolic volumes and enhanced LV relaxation during diastole (24, 25). These studies shine a spotlight on the RV-LV connection in patients with significant TR and underscore the point that LV function, particularly diastolic function, should be viewed with the phenomenon of interventricular interaction in mind. A recent meta-analysis of TTVI studies shows that despite a reduction in echocardiographic measures of RV function, there is an increase in forward stroke volume and cardiac output (26). The results of the currently enrolling randomized trials of various TV devices should enhance our understanding of the relationship between TR reduction and changes in RV and LV hemodynamics, with both patient centered outcomes as well as heart failure hospitalizations and mortality.

Baseline reduced RV function has been associated with poor outcomes following isolated surgical interventions (6). Measures of RV function that predict outcomes may help inform timing of intervention. CMR is considered the gold standard when evaluating RV volumetrics and does not use intravenous contrast, often a limiting factor in TR patients with concomitant renal dysfunction, or use ionizing radiation (27). It offers detailed multiplanar views of all cardiac structures and provides a comprehensive quantitative and qualitative assessment of TR and right heart chamber volumes, mass and ejection fraction. Patients with CIED must be considered as some devices are not CMR-compatible and CIED leads can diminish imaging quality. While CMR lacks robust validation with regards to TR severity grading and prognostication, recent publications have correlated TR severity with outcomes and proposed quantitative parameters of TR severity (28, 29). Contrastingly, RV evaluation by CMR is well-validated as the reference standard (30) and has been shown to be predictive of post-operative mortality after isolated TV surgery for functional TR (31). When combined with two-dimensional echo measured tricuspid annular plane systolic excursion (TAPSE), a CMR-derived RV ejection fraction of ≤45% predicts outcomes following transcatheter intervention (32). Similarly, 3D echo-derived RV ejection fraction ≤45% has been shown to correlate with outcomes after intervention (33). RV speckle-based strain imaging has also been shown to correlate with outcomes in patients with significant TR (34) and may be useful in risk stratification. Because the RV is sensitive to afterload, measures of the RV response to afterload or RV-PA coupling, have also been associated with outcomes. The ratio of TAPSE to PA systolic pressure measured by echocardiography is the most frequently used metric; a high baseline ratio is associated with decreased all-cause mortality (35) and improved survival after TTVI (36). In addition, RV-PA coupling reserve has been associated with better outcomes following TTVI (36).

Invasive measures of arterial afterload also has an important role in management decisions for patients with TR. Right heart catheterisation is essential to determine pre- versus post-capillary PHT, transpulmonary pressures and pulmonary vascular resistance, and right atrial pressures (37). Differentiating between pre and post-capillary PHT may alter management as guidelines have suggested treatment of the primary cause of PHT is indicated in patients with TR (38, 39). In addition, studies have suggested that patients with pre-capillary PHT have worse outcomes compared to those with post-capillary PHT (40). Currently “severe” PHT (typically a PA systolic pressure of >60 mmHg or PVR of >5 WU) is a contraindication for trial enrolment. It is reasonable to perform right heart catheterisation routinely in order to best evaluate this cohort who often suffer from a combination of cardiac and respiratory comorbidities.

Other baseline clinical parameters that may help to predict outcomes after TTVI include kidney and liver function (41). Cardio-hepatic syndrome was present in 45% of patients with TR and is a strong independent predictor for mortality and heart failure hospitalizations at 1-year follow-up (42). The Model for End-Stage Liver Disease (MELD) is a metric of the degree of hepatorenal disease predicts outcomes in patients undergoing isolated TV surgery (43). Beyond survival benefit, improvement in symptoms, functional capacity and edema remain important considerations in this population with recurrent utilisation of New York Heart Association (NYHA) class, 6-min walk distance (6MWD), edema scores and Kansas City Cardiomyopathy Questionnaire (KCCQ) assessments to measure pre-morbid function and subsequent clinical response. As numbers treated for TR continue to increase, this prognostication should become more reliable, enhancing patient selection and clinical outcomes.

TR is a dynamic condition that, depending on its mechanism, varies depending on loading conditions related to volume status. A volume-overloaded patient will result in a more dilated tricuspid annulus, more leaflet tethering and a broader coaptation gap. Performing TEE or CCT imaging evaluation in this state will reduce the likelihood of suitability for some if not all therapies. In many patients, admission for volume optimisation under the care of a multidisciplinary Heart Team, often spearheaded by an advanced heart failure service, is vital both in the evaluation phase and again pre-procedure. This “pre-habilitation,” often further guided by invasive right heart catheterisation hemodynamics, should increase likelihood of meeting anatomical criteria required for device intervention and assist with procedural success. “Pre-habilitation” should be strongly considered for any patient both prior to evaluation for intervention and again in the days prior to their procedure.

## Transcatheter therapies

**Figure 3:**
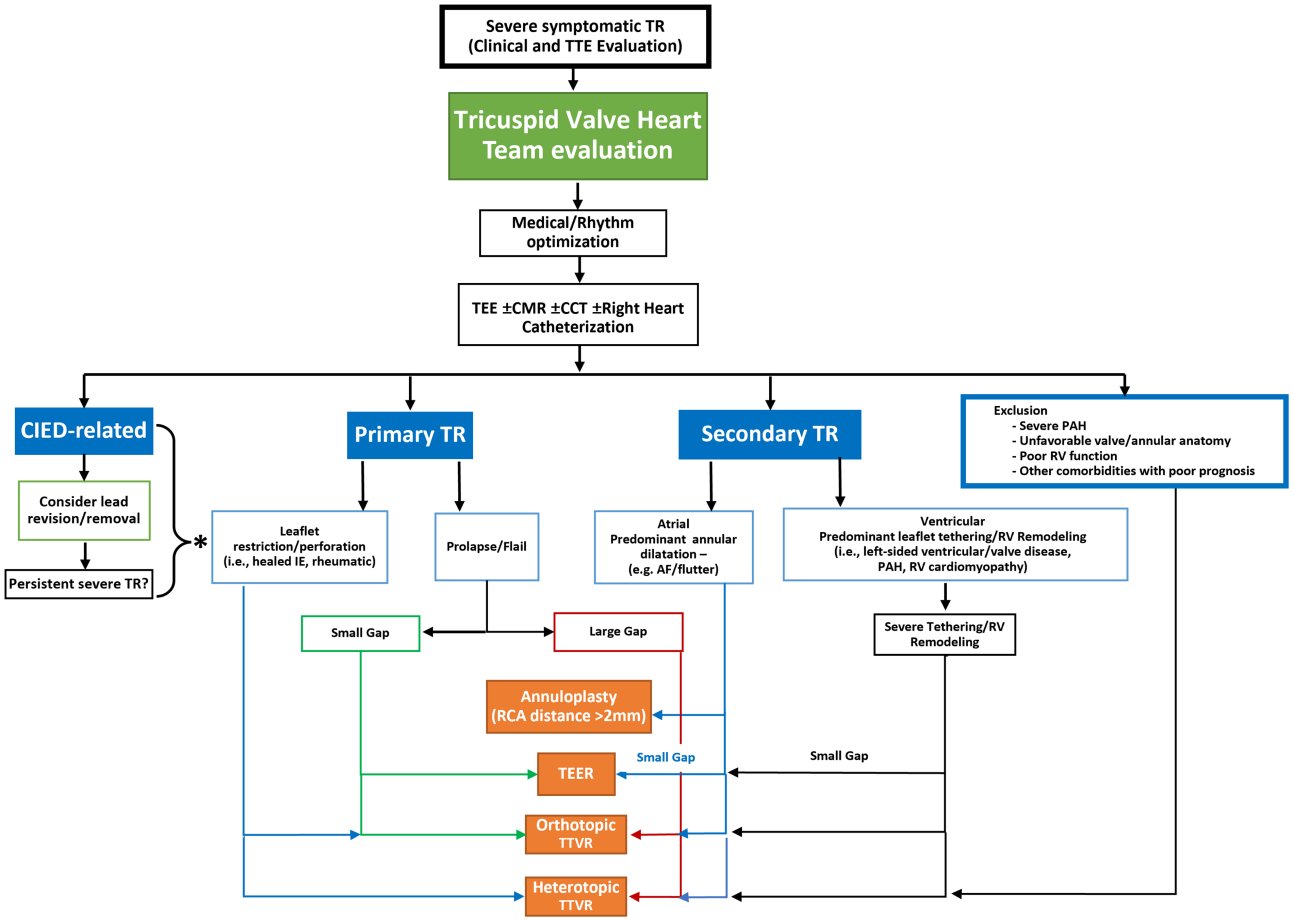
Proposed anatomic algorithm for the selection of transcatheter tricuspid valve intervention systems. Following a determination of the presence of severe, symptomatic tricuspid regurgitation (TR), the patient should be referred to a heart team with experience in management of TR. Patients should be medically optimized which may entail rhythm management. If TR persists, then a more comprehensive imaging or invasive assessment may be required to determine the etiology and morphology of the disease. Once this is determined the anatomic characteristics of the disease may determine the most appropriate transcatheter therapy. *If severe TR persists after lead removal, or the TR is not caused by the cardiac implantable electronic device (CIED) then the etiology of the TR should be assessed to determine the possible device choices. CCT, cardiac computed tomography; CIED: cardiac implantable electronic device; CMR, cardiac magnetic resonance imaging; IE, infective endocarditis; MDT, multidisciplinary team; PAH, pulmonary arterial hypertension; RCA, right coronary artery; TEE, transesophageal echocardiography; TEER, transcatheter edge-to-edge repair; TR, tricuspid regurgitation; TTVR, transcatheter tricuspid valve replacement.

TTVI for TR require careful selection relating to valve anatomy, mechanism of TR, presence of CIED related disease and right heart size and function. These considerations guide therapeutic strategy, repair, or replacement, with techniques that emanate from surgical repair such as leaflet approximation, direct annuloplasty and orthotopic or heterotopic valve replacement (Table 1). A proposed algorithm (Figure 3) outlines some criteria for deciphering between treatment options taking into account the valve pathology, leaflet anatomy, coaptation gap as well as patient factors.

**Table 1:** Considerations when choosing type of transcatheter therapy for tricuspid regurgitation.

	Leaflet approximation	Annuloplasty	Orthotopic valve replacement	Heterotopic caval valve implantation
Primary Etiology	+	**−**	+	+/−
Coaptation Gap > 7 mm	**−**	+/−	+	+/−
Complex Subvalvular Anatomy	**−**	+/−	+/−	+/−
CIED with Impingement	+/−	**−**	+	+/−
Leaflet Tethering >10 mm	+	**−**	+	+/−
Leaflet Morphology (>3)	+/−	+/−	+	+/−
Large Annulus	+	**−**	**−**	+/−
Small RV dimensions	+	+	+/−	+/−
Poor RV function	+/−	+/−	+/−	+/−
No Anticoagulation	+/−	+	+/−	+/−
Difficult TEE Imaging	+/−	+/−	+/−	+/−
Unfavorable IVC anatomy	+/−	+/−	+/−	**−**

+, Favorable; +/−, May or may not be favorable; —, Not favorable.

RV, right ventricle; TEE, transesophageal echocardiography; IVC, inferior vena cava.

### Transcatheter tricuspid valve repair techniques

These techniques include leaflet approximation (i.e., TEER) and annuloplasty devices. Current clinical data of these therapies are summarized in Table 2.

**Table 2:** Summary of available clinical data in transcatheter tricuspid repair.

	TriClip (*n* = 85)	PASCAL (*n* = 34)	Cardioband (*n* = 30)	Trialign (*n* = 15)	FORMA (*n* = 19)	Mistral (*n* = 7)
**Baseline**						
Age, years	77.8 ± 7.9	76.3 ± 10.4	75.2 ± 6.6	73.6 ± 6.6	76 ± 9	76.0
Female	66%	52.9%	73.3%	86.7%	73.7%	57%
EuroSCORE II	8.7 ± 10.7	5.3 ± 5.2	4.1 ± 2.8	-	9.2 ± 5.6	4.0
NYHA III/IV	75%	79.4%	83.3%	66.7%	94.7%	100%
**TR etiology**						
Functional	84%	87.9%	100%	100%	100%	100%
CIED RV lead	14%	11.8%	13.3%	0%	15.8%	14%
<Severe TR	8%	3%	24%	-	5%	0%
**Procedural outcomes**						
Procedural time (mins)	75^a^	167.7 ± 152^a^	254.5 ± 92.8	124 ± 62^a^	-	58–125
Technical success	100%	85.3%	100%	100%	89.5%	100%
In-hospital mortality	0	0	3%	0	0	0
Conversion to surgery	0	0	0	0	5.3%	0
Device embolization	0	0	0	0	5.3%	0
Conduction disturbance	0	0	3%	0	0	0
Coronary complication	0	0	10%	6.7%	0	0
**Follow-up outcomes**						
Follow-up	1 year	30 days	6 months	30 days	32 months	30 days
Mortality	7.1%	0%	10%	0%	23.5%	0
Stroke	1.2%	0%	3%^b^	0%	0%^b^	0
Major bleeding	11.9%	5.9%	13%	0%	10.5%^b^	0
Malposition	6%^c^	2.9%^c^	0	20%^d^	5.3%	0
**TR severity outcomes**						
<Severe	71%	52%	73%	-	67%	-
≥1 grade reduction	87%	85%	-	-	-	100%
PISA EROA, cm^2^ (mean)	0.65 to 0.32; *p* < 0.0001	0.77 to 0.48; *p* = 0.007	0.76 to 0.39; *p* = 0.0004	0.51 to 0.32; *p* = 0.020	0.92 to 0.77; *p* = 0.52	0.52 to 0.15^e^; *p* < 0.01
Regurgitant Volume (ml/beat)	52.20 to 27.68; *p* < 0.0001	51.52 to 38.80; *p* = 0.060	87.4 to 49.5; *p* = 0.036	79.6 to 57; *p* = 0.065	-	49.4 to 19.7^e^; *p* < 0.01
Vena contracta (cm)	1.73 to 0.78; *p* < 0.0001	1.50 to 0.78; *p* < 0.001	1.20 to 0.88; *p* < 0.0001	1.3 to 1.0; *p* = 0.022	1.18 to 0.84; *p* = 0.005	0.95 to 0.62^e^; *p* < 0.05
**Right heart remodeling**						
RV base end diastolic dimension (cm)	5.28 to 4.79; *p* < 0.0001	3.86 to 3.60^f^; *p* < 0.05	3.76 to 3.72^f^; *p* = 0.71	-	5.54 to 5.2; *p* = 0.19	-
Tricuspid annular diameter septal-lateral (cm)	4.34 to 4.03; *p* < 0.0001	4.51 to 4.27; *p* = 0.015	4.16 to 3.78; *p* = 0.0014	4.0 to 3.8; *p* = 0.038	4.6 to 4.3; *p* = 0.090	-
TAPSE (cm)	1.44 to 1.59; *p* = 0.0002	1.45 to 1.70; *p* = 0.39	-	1.7 to 1.6; *p* = 0.31	1.53 to 1.48; *p* = 0.80	1.6 to 2 (median); *p* < 0.05
**Clinical outcomes**						
NYHA I/II	83%	83%	88%	100%	66.7%	100%
6MWD	+31 m *p* = 0.002	+70 m *p* < 0.001	+60 m *p* = 0.004	+53 m *p* = 0.008	+54 m *p* = 0.016	+102 m p < 0.05
KCCQ	+20 *p* < 0.0001	+15 *p* < 0.001	+24 *p* < 0.0001	−26.5^g^ *p* < 0.001	+16.2 *p* = 0.016	+20.2 *p* < 0.05

a Implant time.

b 30-day follow-up.

c Single Leaflet Device Attachment (SLDA).

d Single pledget detachment, no reintervention.

e Medial.

f RV mid end diastolic dimension.

g MLHFQ, Minnesota Living with Heart Failure Questionnaire.

NYHA, New York Heart Association; CIED, Cardiac implantable electronic device; RV, Right ventricle; TR, Tricuspid regurgitation; PISA, Proximal isovelocity surface area; EROA, Effective regurgitant orifice area; TAPSE, Tricuspid annular plane systolic excursion; 6MWD, 6 min walk distance; KCCQ, Kansas City Cardiomyopathy Questionnaire.

#### Leaflet approximation devices

Tricuspid TEER is the most utilized device worldwide, with the initial off-label use of the MitraClip system (Abbott Vascular, Santa Clara, CA, USA) before the development and CE mark approval of the PASCAL (Edwards Lifesciences, Irvine, CA, USA) and TriClip (Abbott Vascular, Santa Clara, CA, USA) devices. Detailed pre-procedure TEE evaluation of the mentioned valve characteristics can assist with determining procedural suitability and likelihood of success using edge-to-edge repair techniques.

TV leaflet morphology, number, and location (9), influences procedural strategy, clip position, number of clips and likelihood of success (10, 44). Certain characteristics that correlate with successful TR reduction include short coaptation gap, central or anteroseptal site of main TR jet and 3-leaflet TV morphology. Using older generation clip devices, large coaptation gaps of ≥7.0 mm, were associated with residual severe TR at end of procedure (45, 46) although with longer devices, a wider gap of 8.5 mm is treatable (47). Non-central or non-anteroseptal TR jets also significantly reduce success rates (10). Approximating a lateral leaflet to the septal leaflet reduces annular dimensions which promotes favorable remodelling and has been associated with improved cardiac output in flow models (48). A low leaflet-to-annulus index, the ratio of the sum of the anterior and septal tricuspid leaflet length in relation to the septolateral tricuspid annulus, is correlated with worse procedural success (46) higher likelihood of failure to achieve an anteroseptal clip placement or failure to place any device. Leaflet morphology, including number of leaflets, has become an important consideration when evaluating for leaflet approximation.

Other considerations that may influence patient selection for the TEER devices include baseline RV dysfunction, CIED related TR and contraindication for anticoagulation. Because an increase in RV afterload following TTVI is directly related to the amount of TR reduction the TEER devices may offer some advantages to the patients with baseline severe RV dysfunction where more modest TR reduction may not result in abrupt increases in afterload. However, this remains a theoretical advantage since no data supports differences in RV function following different devices. CIED leads can interfere with leaflet approximation and reduce TEER efficacy in cases where the lead is impinging on the leaflet and directly causative of regurgitation. In such scenarios, it is reasonable to consider lead removal/revision and an alternate CIED such as coronary sinus lead implantation or leadless devices (e.g., Micra device). Unfortunately, the effectively of lead removal remains questionable and likely related to the duration of implantation, and the actual mechanism of lead impingement (49, 50). One must also consider safety of leaflet approximation in patients with new CIED implants as there can be interaction and dislodgement. These considerations should factor into decision-making, understanding the patient risk and goals of treatment when determining strategy.

##### Triclip (previously off-label MitraClip)

###### Device and procedural aspects.

TriClip guide catheter is different from the MitraClip, having septal to lateral motion capability and a shorter guide curve. TriClip (Abbott Vascular) edge-to-edge repair is routinely performed under general anesthesia and TEE-guided with 24Fr guide sheath and introducer inserted *via* the common femoral vein and IVC over a guidewire. The TriClip delivery system is made up of a delivery catheter and an implantable clip that varies in size by length and width as used in MitraClip procedures; NT (standard length and width), NT-W (extra wide), XT (extra long) and XT-W (extra long and wide). An individualized approach is used to determine the appropriate device size.

###### Current outcomes data.

In the TRILUMINATE trial of 85 patients evaluated at one-year follow-up, TR severity was reduced by at least one grade in 86% of patients (51). Moderate or less TR was achieved in 70% of patients and, importantly, in 56% of those with massive or torrential TR. Significant improvements in echocardiographic (VC width, EROA, regurgitant volume and PISA radius, RV end diastolic diameter) and clinical parameters (NYHA class, 6MWD, KCCQ) were observed, with the achieved 30-day results maintained at one-year. There was an 8% rate of single leaflet device attachment but no device embolization. Edge-to-edge repair has shown to improve left ventricular filling and stroke volume on follow-up CMR imaging (52). This is likely due to the net improvement in RV effective forward flow primarily due to reduction in regurgitant volume and was shown to correlate with improved NYHA functional class, 6MWD and reduced peripheral edema.

##### Pascal

###### Device and procedural aspects.

The PASCAL repair system (Edwards Lifesciences) includes a 22Fr transfemoral guide sheath with a device consisting of a central spacer connected to two broad paddles with independently moving clasps. The device is made of nitinol, and uses a passive closure system that may reduce leaflet injury and stress. The PASCAL delivery system and device are the same for both mitral and tricuspid TEER procedures. The central spacer is helpful in occupying the regurgitation orifice and reducing leaflet stress. The newer PASCAL Ace implant features a narrower and shorter profile with longer clasps compared with the original PASCAL implant, all tailored to manage complexities of tricuspid repair; notably large TV annulus, dense chordae structures and wide coaptation gaps. In scenarios where the repositioning above the leaflets is desired, the device it can be elongated and retracted across the valve without leaflet/subvalvular injury or entanglement.

###### Current outcomes data.

The early feasibility CLASP TR study demonstrated safe profile with 85% TR reduction of at least one grade in 29 patients who had successful implantation (53). Five other patients were attempted and devices were retrieved without implantation due to complex anatomy. 32% had two implants with mean time from first implant release to final implant of 168 ± 152 min. One patient had single leaflet device attachment. At 30 days there was TR improvement with 52% at moderate or less as well as significant improvement in EROA by PISA and mean vena contracta. RV end diastolic diameter significantly reduced while LV stroke volume index significantly improved and improvements in functional status, exercise capacity and quality-of-life measures were significant.

##### Mistral

###### Device and procedural aspects.

This spiral-shaped nitinol device improves leaflet apposition by gathering targeted chordae together atraumatically, forming a “flower bouquet” shape that reduces the coaptation gap through chordae and leaflet approximation. This transfemoral procedure involves an 8.5Fr steerable sheath that directs a delivery system with the Mistral device (Mitralix, Yokne’am, Israel) into the RV. The device comes in either 8.8 mm outer diameter or a 7.4 mm outer diameter with both the same inner diameter of 5 mm. It engages leaflet chordae at the level between papillary muscles and leaflet tips and is rotated clockwise for a total of five full circles. This can be undone by reversing rotation to optimise chordae grasp. Stability tests are done throughout the various intervals of the procedure including before deployment.

###### Current outcomes data.

Seven cases are reported with procedure time 58 to 125 min. All procedures were successful with 30-day outcomes demonstrating significant TR reduction, symptomatic benefit and no adverse events (54).

#### Annuloplasty devices

Annuloplasty, a common additive intervention for patients requiring cardiac surgery, is yet to establish itself as a transcatheter therapy. Being an annular treatment, it conceptually addresses the pathology of functional TR due to annular dilatation more so than leaflet approximation. To achieve surgical-like annuloplasty results, most transcatheter devices extend from anteroseptal to posteroseptal commissures to form an incomplete ring that spares the septal leaflet and atrioventricular node. This addresses annular dilatation in the anteroposterior direction which is what occurs in functional TR. Additionally, annuloplasty has the benefit of preserving native anatomy, allowing for future intervention with leaflet modification or valve replacement and does not require anticoagulation. Transcatheter annuloplasty systems can be grouped into ring (direct or indirect), suture and non-suture-based devices.

Overall, there appears to be adequate procedural safety and efficacy in early feasibility studies but there are several notable procedural challenges and novel complications. Procedural imaging is again crucial and can be difficult in these procedures. Many direct annuloplasty devices require sequential annular anchoring, a meticulous and time-consuming process that when combined with difficult imaging, results in very long procedural times averaging nearly 5 hours (55). Impingement of important cardiac structures is seen in these procedures due to their proximity to the tricuspid annulus. At-risk structures include the RCA, the conduction system (atrioventricular node and right bundle of His) coursing the membranous septum near the anteroseptal commissure and the coronary sinus ostium near the posteroseptal commissure. RCA course is evaluated by CT pre-procedure with a high rate of patients meeting exclusion criteria due to proximity and risk of impingement (56). Furthermore, coronary angiography is important to rule out significant disease and ensure safe coronary wiring of the vessel to not only delineate its location during the procedure but serve as protection. Even so, there is still a 15% risk of RCA perforation or complication requiring stenting in one study of 60 patients undergoing Cardioband (57). In the context of prolonged procedure times and risk of impinging cardiac structures, early results are encouraging and appear to offer similar benefit to leaflet repair techniques. The majority of devices are direct, ring-based annuloplasty devices with a few early investigational indirect annuloplasty systems.

##### Cardioband

###### Device and procedural aspects.

The first generation Cardioband Tricuspid Repair System (Edwards Lifesciences) is a direct, sutureless, and adjustable surgical-like Dacron band based on the mitral valve system. Guided by TEE (and ICE when required), an implant catheter is inserted through a 24Fr transfemoral access sheath and rested on the atrial side of the tricuspid annulus, where up to 17 anchors are deployed on the atrial side along the anterior annulus from the anterolateral commissure to the posteroseptal region. The band is sized according to pre-procedural CT measurements, with five different sizes dictating the overall number of anchors. For safety, a guidewire is placed down the RCA to provide a fluoroscopic landmark of its course. At each anchor implant, position should be carefully confirmed on echocardiography with a pull test to ensure secure insertion. Once all anchors are deployed, the implant delivery system is detached from the band and removed. The retained wire is then used to pass the size adjustment tool that attaches to the spool. Bidirectional reshaping of the annulus is then possible using the pre-mounted contraction wire that is connected to the adjusting spool. The device is then cinched in a controlled fashion with live visualisation of annular reduction and TR improvement. The band is contracted between 3.5–5.5 mm depending on size and echocardiographic findings. Then the device is released and the system is removed.

###### Current outcomes data.

The six-month and then two-year outcomes of the first-in-human prospective trial were reported totalling 30 patients (58, 59). There was 100% technical success with mean procedure time of 254 ± 93 min. There were four adverse events relating to injury of nearby cardiac structure (three RCA complications, 1 atrioventricular block). There was statistically-significant reduction in septolateral annular diameter of 16%. 72% of patients maintained less than severe TR and 82% of patients were in NYHA Class I-II. 6MWD and KCCQ score also improved. The early feasibility trial (NCT03382457) was temporarily paused to iterate the device and has since been restarted with a second generation device.

##### DaVingi TR system

###### Device and procedural aspects.

The DaVingi TR system (Cardiac Implants, Wilmington, Delaware) is a direct annuloplasty system that is implanted in two stages. An annuloplasty ring is passed through a 22Fr catheter from the jugular vein and positioned on the annulus from the atrial aspect. Anchors fix this to the annulus by being simultaneously fired. An adjustment connector is left fixed to the jugular vein and, 3 months later when the ring has healed, a second procedure is done to contract the annular ring size. This study is still recruiting with successful first-in-human experience published (60).

##### Transatrial intrapericardial tricuspid annuloplasty (TRAIPTA)

###### Device and Procedural Aspects.

Transatrial intrapericardial tricuspid annuloplasty is an indirect, fully retrievable, transpericardial annuloplasty system delivered within the pericardial space *via* a right atrial appendage puncture. An adjustable lasso-like implant is opened and loops around the apex of the heart until it rests within the atrioventricular groove. Then the delivery system is removed and the device is tightened using a sliding Roeder knot, exerting external pressure at the tricuspid and mitral annular level. The right atrial appendage puncture is then closed using a nitinol occluder. A study in 16 swine demonstrated significant TV geometry changes (reductions in annular area and perimeter with improved TV coaptation) with minimal impact on the mitral annular geometry and hemodynamics (61). Patients with previous pericardial adhesions or pericardiotomy would not be eligible for this therapy. Further developments for human use are pending.

##### Minimally invasive annuloplasty (MIA) device

###### Device and procedural aspects.

Minimally invasive annuloplasty (MIA) technology (Micro Interventional Devices) consists of low-mass, polymeric, self-tensioning PolyCor anchors and the thermoplastic MyoLast polymer for tensioning of the anchors. A series of PolyCor anchors are placed along the posterior annulus and tension is applied to the attached suture which is then locked, plicating the annular tissue. Surgical feasibility in three patients was proven in the Study of Transcatheter Tricuspid Annular Repair (STTAR) trial with good safety and mean 43% reduction in tricuspid annulus area achieved (62).

###### Current outcomes data.

The 12Fr transcatheter system is under trial currently with initial 31-patient experience presented demonstrating reduction in annular area and TR grade as well as improved quality of life with a 10% pericardial effusion rate but otherwise good safety outcomes (63).

##### Pledget-assisted suture tricuspid annuloplasty (PASTA)

###### Device and procedural aspects.

This is another pledget-assisted suture tricuspid annuloplasty (PASTA) technique that involves traversing both the septal to lateral annulus and exchanging for two pledgeted sutures, each with two insertions to maximise force as a point of difference. Approach is transjugular with a deflectable catheter just like TriAlign or alternately can be transapical. The sutures are then tightened using a Cor-Knot device (LSI Solutions). Feasibility has been shown in 22 pigs (64). The double-puncture pledget technique is thought to bicuspidize the TV with more pull-through force which conceptually may lead to better TR reduction but may also lead to more suture dehiscence. There are no human studies currently.

##### Transcatheter Alfieri stitch for tricuspid insufficiency (TASTI)

###### Device and procedural aspects.

This is a transapical leaflet traversal technique of similar principles to TriAlign, whereby the septal and lateral leaflets are each traversed with guidewires that are then snared and exchanged for sutures with pledgets on RA side *via* an additional jugular venous access. Then, pledgets are secured with Cor-knot to create double valve orifice and reduce TR. This has been performed in humans but no literature has been published as yet (65).

##### K-clip

###### Device and procedural aspects.

The K-Clip transjugular system (Huihe Medical Technology, Shanghai, China) involves an outer deflectable sheath that is placed in the right atrium and through this, an inner deflectable sheath with a clip is inserted. The clip has a central corkscrew which is screwed into the annulus to a max depth of 4 mm in a position that is parallel to the annulus, between the leaflets and a wire-protected RCA, and aiming towards the anteroposterior commissure. Then the clip is deployed, shortening the circumference of the tricuspid annulus whilst ensuring no coronary stenosis with selective angiography. The clip is then released and sheaths withdrawn. In 18 pigs, there was 100% procedural success with significant annular area reduction and without major complication (66). Procedural time was notably short at 23.7 ± 4.2 min. There are no cases reported in humans.

### Coaptation enhancement devices (spacers)

These devices are designed around placement of a central spacer that enhances leaflet coaptation and reduces the regurgitant orifice area. The spacer is attached to a second component, either an anchoring caval stent or an anchor secured within the heart. The perceived benefits of this device type include that they can accommodate a large range of annular sizes and extreme coaptation gaps, a current limitation of most transcatheter tricuspid interventions. They also avoid direct interaction with annular structures, may not require complex imaging and may have short device times when compared to other types. However, this would not allow for re-intervention and more clinical outcome data is still required.

#### CroiValve

##### Device and procedural aspects

CroiValve DUO (CroiValve, Dublin, Ireland) is a coaptation spacer valve attached to a superior vena cava (SVC) stent anchor system that has fewer anatomical contraindications with a single size of valve and delivery system for all anatomy. The system is delivered from the internal jugular vein with an adjustable, steerable catheter. The spacer is anchored to a deployed SVC stent and is not in contact with the tricuspid annulus or nearby cardiac structures. It may accommodate anatomical variability and very large annular sizes up to 65 mm. It is planned for further human testing this year.

#### Tripair

##### Device and procedural aspects

Tripair (Coramaze Technologies, Tikva, Israel) consists of a central spacer with a flexible center column attached to an atraumatic right atrial crown that rests in the right atrium. This is implanted transfemorally, can be deployed quickly without complex imaging and is fully retrievable. It has yet to be used in humans.

### Transcatheter tricuspid valve replacement (TTVR) systems

TTVR is establishing itself as a less-invasive valve replacement that, unlike leaflet approximation and annuloplasty, aims to abolish TR. Technical considerations relate to mechanism of anchoring (leaflets vs. annulus vs. chords), annulus shape/size, delivery approach and IVC course to the annulus. Replacement may theoretically be harmful in certain patients with poor RV reserve however how to measure RV function and reserve are areas of continued research. The IVC approach and angulation relative to the tricuspid annulus can influence device delivery, limit coaxiality and impact procedural success. Different TTVR systems have different anchoring mechanisms and thus different imaging requirements. In general, these intraprocedural imaging requirement are less for TTVR than for leaflet approximation or annular devices.

The device characteristics and mechanisms of deployment are discussed in Table 3. TTVR devices may be implanted in patients with large coaptation gaps, restricted leaflets or CIED-related TR. Notable however, the CIED lead is entrapped between the device and native tissue making removal more difficult. Anticoagulation is currently recommended given the increased likelihood of valve thrombosis in a low pressure right-sided system with additional antiplatelet therapy considered. Current devices sizes address limited annular dimensions, another major drawback of TTVR at this time.

**Table 3:** Summary of orthotopic and heterotopic tricuspid valve replacement device characteristics.

	EVOQUE	NaviGate	LuX valve	TriSol	Intrepid	TricValve	Tricento
Manufacturer	Edwards Lifesciences	NaviGate Cardiac Structures	Jenscare Bio-technology	TriSol Medical	Medtronic	Products + Features	New Valve Technology
Frame and Design	Nitinol frame with fabric skirt, nine anchors	Cone-shaped nitinol stent with 12 tines and 12 atrial winglets	Nitinol stent with atrial disc, interventricular septal anchor, two graspers	Cone-shaped nitinol frame with six fixation arms	Dual-stent self-expanding nitinol	Two self-expanding nitinol stents	Self-expanding nitinol
Pericardial Leaflets	Bovine	Equine	Bovine	Bovine	Bovine	Bovine	Porcine
Components	1	1	1	1	1	2	1
Anchoring	TV leaflets/ annulus	TV leaflets/ annulus	Septal anchor and anterior leaflet grasp	Tricuspid annulus	Perimeter oversizing	Stent expansion/ radial force with device oversizing	Stent expansion/ radial force with device oversizing
Radial Force	Dependent	Dependent	Independent	Dependent	Dependent	Dependent	Dependent
Sizes (mm)	44,48,52	36,40,44, 48,52	50,60,70 (annulus)	62.5 (outflow) 50.3 (inflow)	43,46,50	Up to 38 (SVC) Up to 43 (IVC)	Up to 48
Delivery	Femoral	Jugular / Atrial	Atrial / Mini-thoracotomy	Jugular	Femoral / Apical	Femoral	Femoral
Recapturable	No	No	No	Yes until fixations arms fully expanded	Yes	Yes (to 80% deployed)	No
Delivery system size (Fr)	28	42	32	30	35	27.5	24

Fr, French size; SVC, superior vena cava; IVC, inferior vena cava.

#### EVOQUE

##### Device and procedural aspects

The EVOQUE system (Edwards Lifesciences) is a 28Fr transfemoral venous system which can allow for a 44 mm, 48 mm or 52 mm diameter valve implant. The delivery system is capable of primary and secondary flexion as well as adjustable depth. The valve is made of bovine pericardium, is trileaflet, has a nitinol frame with nine anchors and a fabric skirt. After positioning of a pre-shaped guidewire carefully toward the RV apex and in a central position across the TV, the delivery system is advanced to the RA where it is flexed across the TV. After advancement of the delivery capsule below the valve, position and trajectory are optimised and anchors are exposed below the leaflets but above the papillary muscle heads. Then as the valve is exposed and expands, the anchor tips become positioned subannular to capture the leaflets. When there is adequate leaflet capture and positioning below the annulus, the valve is fully deployed and released, with careful system removal without interaction with the valve.

##### Current outcomes data

The TRISCEND study (Edwards EVOQUE TV replacement: Investigation of Safety and Clinical Efficacy after Replacement of TV with Transcatheter Device) 30-day results of 56 patients demonstrated mild or less TR in 98% with improvement in NYHA class, 6MWD and Kansas City Cardiomyopathy Questionnaire (67). Median device time (implant insertion to release) was 70.1 min. The composite major adverse events rate was 26.8% at 30 days caused by one cardiovascular death in a patient with a failed procedure, two reinterventions after device embolization, one major vascular complication and 15 severe bleeds, of which none were life-threatening or fatal. 11.1% required permanent pacemaker implantation.

#### LuX valve

##### Device and procedural aspects

The LuX valve (Jenscare Biotechnology, Ningbo, China) is a 32Fr flexible delivery system with a bovine pericardial valve on a nitinol valve stent that has an atrial disc, an interventricular septal anchor “tongue” and two expanded polytetrafluoroethylene-covered graspers. It has four valve sizes (30 to 55 mm) and eight disc sizes to treat annular diameters of 25 to 50 mm. It is delivered *via* mini right thoracotomy or transatrial approach and is radial force-independent unlike other TTVR devices. This is designed to minimise force-related complications of conduction disturbance and RCA impingement but may be at a trade-off for increased risk of paravalvular regurgitation.

##### Current outcomes data

Procedural success was achieved in 45 of 46 cases with one fatal RV perforation. Mean procedure time was 150 min. 33 patients achieved none or mild TR at six months follow-up (68).

#### TriSol

##### Device and procedural aspects

TriSol (TriSol Medical, Yokneam, Israel) is a bovine pericardial monoleaflet valve attached to two commissures to create a bicuspid prosthesis. This is mounted on a thin, self-expanding conical nitinol frame with an inner waist and six circumferential fixation arms that engage and anchor the valve between the native leaflets and the adjacent walls. It is delivered *via* transjugular approach *via* a 30Fr sheath. It is collapsible and repositionable until the fixation arms are fully expanded. It can be used in a septal-lateral annular diameter of 40 to 53 mm. There is one published case report of its successful use (69).

#### Intrepid

##### Device and procedural aspects

Intrepid (Medtronic Inc., Minneapolis, MN, USA) is a circular inner stent that houses a trileaflet bovine pericardial valve and is delivered *via* either transapical or transfemoral access *via* a 35Fr delivery system. It is designed for mitral and tricuspid intervention. It features the ability to perform multidirectional steering and has a unique atrial-to-ventricular deployment. An early feasibility study is continuing recruitment (NCT04433065).

#### Other

Other TTVR devices are within preclinical and FIM studies. They include TriCares (Aschheim, Germany, NCT05126030), VDyne (VDyne Inc., Maple Grove, Minnesota), and CardioValve (Valtech, Yehuda, Israel, NCT03958773).

### Heterotopic caval valve implantation

Caval valve implantation is considered an alternative option as a palliative treatment where direct tricuspid intervention, transcatheter or surgical, is deemed unsafe, unsuitable or is not available. Such scenarios include patients with an enormous tricuspid annulus and large coaptation gap rendering TTVR or leaflet approximation impossible. Likewise some patients with carcinoid heart disease develop TV thickening and dysfunction due to fibrotic plaque deposition at the leaflet tips, making leaflet approximation challenging. Conceptually, caval valve implantation (unicaval or bicaval) aims to reduce venous congestion. It can be performed using a dedicated implant (TricValve, Tricento) or utilising a balloon-expandable valve with pre-stent implantation for anchoring. Imaging requirements are minimal and the devices may be implanted using fluoroscopic imaging and contrast angiography.

#### TricValve

##### Device and procedural aspects

TricValve (Products + Features, Vienna, Austria) is a 27.5Fr transfemoral system with two self-expanding caval valves. The SVC valve has a central belly to better prevent dislodgement, a long skirt to reduce paravalvular leak and an uncovered superior segment to allow innominate vein flow. The inferior vena cava (IVC) valve is of higher radial force to support fixation and has a short skirt to avoid hepatic vein obstruction. SVC stenting is performed with a stiff wire from the right femoral vein to either the right subclavian or internal jugular vein. Then valve deployment should start high so that there can be some gentle downward device traction to improve stability and is recapturable to 80% deployed. For IVC deployment, the IVC stent has a short covered section and it’s superior aspect should be near the RA junction. The valve is deployed again starting superior with downward traction to land between the RA and the hepatic vein. Imaging considerations to ensure anatomic suitability include sequential anteroposterior measurements of the SVC (maximum 34 mm) and IVC (maximum 43 mm), length of SVC to middle of perpendicular right PA and SVC-to-RA length and length of IVC from RA junction to hepatic veins (at least 10 mm). SVC stenting can be done in patients with CIED with leads trapped behind the stent. This device avoids TV/RV anatomical exclusions and can be fluoroscopically-guided avoiding general anesthesia. However it does require intraprocedural contrast angiography.

##### Current outcomes data

The TRICUS EURO study of thirty-five implants showed 94% procedural success with one SVC prosthesis migration without embolization (70). At six months follow-up, there were no significant differences in echocardiographic parameters other than reduction in presence of hepatic vein backflow. KCCQ, NYHA class and loop diuretic dose reduction was significant but there was no difference in renal function or liver enzymes and a significant increase in NT-proBNP.

#### Tricento

##### Device and procedural aspects

Tricento (New Valve Technology, Hechingen, Germany) is a 24Fr transfemoral system that has a long self-expanding nitinol stent frame designed for the custom length from superior SVC to above the hepatic vein. Within this is a lateral bicuspid pericardial tissue valve that is orientated to be facing the RA on deployment. This single device implant can accommodate pre-screened diameters of 16 to 35 mm for the vena cavae, 40 to 80 mm RA length and at least 10 mm distance from RA to hepatic vein. This system can accommodate existing CIED leads and can be done without general anesthesia but does require contrast angiography.

##### Current outcomes data

21 patients were analyzed with 100% procedural success at mean total procedure time and device time of 92 ± 48 min and 20 ± 7 min, respectively, (71). NYHA class improvement was seen and echocardiographic parameters were not different at median 93-day follow-up. In seven patients with pre and post CMR imaging, RV end-diastolic volume was reduced from 252 ± 65 mm^3^ to 221 ± 46 mm^3^ (*p* value = 0.018), an interesting finding albeit in a small cohort. Three patients had stent fracture detected without valve compromise and were thought to be associated with massive TR with high mechanical stress during systole applied to the stent segment facing the caudal valve frame.

#### Inferior caval valve implantation (CAVI)

##### Device and procedural aspects

CAVI is a somewhat simplified and commercially-feasible option for patients being considered for palliative caval valve implantation. This involves transfemoral balloon-expandable valve implantation (e.g., An Edwards Lifesciences SAPIEN valve) within a self-expanding IVC stent. One study comparing CAVI to medical therapy demonstrated only short-term improvement in dyspnea that did not persist and high incidence of both procedural complications and valve/stent migration culminating in early termination of the trial (72). This technique appears to be superseded by the dedicated systems aforementioned and should be considered with some caution.

## Post-procedural management

Post-procedural care in this heterogenous population must be tailored to the patient and the procedure. While there are broad approaches to managing this patient group, it should be patient-specific based on medical history, volume status, type of intervention performed and its success, residual TR and RV function and need for/timing of anticoagulation. Diuresis is generally continued, particularly after non-replacement therapies, with thorough evaluation of how the RV has tolerated the intervention and monitoring of urine output and renal function. Hemodynamic compromise is more common post TTVR where TR is suddenly eliminated and RV afterload is increased. It is not uncommon to continue inotropic support post-procedure and is often required for several days to allow steady volume optimisation and early RV reverse remodelling with support. In contrast, there are patients who tolerate leaflet approximation and are suitable for discharge day one post-procedure assuming no procedural injury.

Currently, anticoagulation is recommended post TTVR which should be initiated as early as safely possible, considering bleeding risks related to the patients history, access-site and even TEE-related gastrointestinal bleeding. Anticoagulation is often already indicated in this cohort who often have a separate indication, commonly for atrial fibrillation. All-cause major bleeding is still a common occurrence in this cohort of significant morbidity, at an incidence of 10%–15% across different platforms (51, 73, 74). With regards to procedural risk across all therapies, gastrointestinal injury including bleeding can occur and is associated with longer procedure time and inferior imaging quality (75). Vascular injury and access-site bleeding rates are exceedingly rare (53). Additional antiplatelet therapy is also a consideration for prevention of leaflet thrombosis.

The incidence of new pacemakers following surgical TV repair may be as high as 14% (76) and so conduction disturbances following TTVR or transcatheter annular repair may be expected. Monitoring following device placement is routine however the duration of monitoring to detect device related conduction disturbances is currently unknown. Complications related to prior CIED devices has not been widely reported however it is reasonable to interrogate device function after TTVI.

## Future

Given the large unmet need by the previous undertreatment of TR, and the relative safety of TTVI from early studies, the landscape of transcatheter TV interventions is rapidly expanding. In this setting our understanding of the disease pathophysiology and our ability to evaluate, both with non-invasive and invasive tools, has also advanced. These assessment tools may allow for greater standardisation of TV disease definitions (7), severity criteria (17) and clinical endpoints (26, 77). Adaptation of TV nomenclature classification with identification of TV morphology variants (9) has been important in this space, particularly given the worldwide surge in leaflet approximation case numbers. The new five-grade TR severity scale of mild, moderate, severe, massive and torrential (17) has been embraced across the major tricuspid therapy trials and is now included in the European guidelines (8, 39). For patients being considered for TTVI, the extended grading scheme and may allow for better adjudication of procedural appropriateness and the assessment of the impact of a single TR grade reduction in improvement of clinical symptoms, exercise capacity and repeat heart failure hospitalisation. Furthermore, it allows for clearer arbitration of degree of benefit across device platforms and will likely ensure a reliable evidence-based approach can be applied to TR patients.

TV intervention is still somewhat in its infancy and as utilisation and familiarity increases, questions relating to device selection and durability continue to be studied. Clarity regarding best device with regards to comparative safety and clinical outcomes will continue to improve with more published data and analysis. Even though the older population currently being treated is often of high-morbidity with poor life expectancy, transcatheter interventions should aim to replicate surgical interventions with regards to longevity and freedom from re-intervention. Concomitant transcatheter treatment of TR at the time of non-tricuspid interventions, attempt to mimic surgical management of multi-valvular disease, however the advantage to transcatheter device therapy is the ability to stage these therapies and possibly reduce unnecessary procedures (78).

Device companies continue to innovate based on real and perceived limitations of current devices. Two novel examples are TriFlo (TriFlo Cardiovascular), a novel three-anchor annular device with a central “mini-valve” and the CroiValve DUO (CroiValve, Dublin, Ireland), a coaptation spacer valve attached to an SVC stent anchor system that has fewer anatomical contraindications. These devices theoretically can offer treatment to extreme annular anatomies and coaptation gaps, a major limitation across current platforms. TV device therapies will continue to evolve given the anatomical limitations of many of the current therapies prohibiting treatment in many patients.

From a procedure perspective, there are some notable drawbacks that are similar to struggles that existed early in aortic and mitral interventions. First, procedural times are long but with familiarity and experience with these techniques and systems, times appear to be improving. However, clearly the anatomy of the TV and related structures is very complex, and currently the reliance on TEE for procedural guidance requires both proceduralist and imaging operator experience and expertise. ICE has an established role in structural intervention and its imaging improvements, with new 4D-capable probes may reduce the imaging intensity as well as imaging-related complications of TTVI. Besides imaging device improvements, device companies will need to continue improving on device safety (i.e., smaller delivery systems) and operator usability much like the advancements seen in the transcatheter aortic devices. Further improvements in delivery systems that allow for responsive multiplanar flexion capabilities will assist with anatomical difficulties including IVC-to-tricuspid annulus offset (Figure 2).

The lifetime management of patients with valvular heart disease must also be considered, as earlier intervention becomes a reality. Procedural “repeatability” and “durability” will start to play a role in TV interventions as it has in TAVR. TTVR and annuloplasty therapies will theoretically allow for a valve-in-device procedures. Removing TEER devices has become a reality with the recent report of mitral TEER electrosurgical laceration followed by transcatheter mitral valve implantation for persistent mitral regurgitation (79).

Structural heart disease management of TR continues to evolve but without early diagnosis and referral, outcomes may not improve. Education of the primary care physician and primary cardiologist in the identification of the symptomatic TR patient and the appreciation of the adverse outcomes associated with even mild disease, (80) is imperative. Referring these patients to Level 1 Valve Centers (38) will allow for appropriate diagnosis of severity, optimization of medical therapy, and access to surgical intervention or trials for TTVI.

## Conclusion

TTVI is expanding at a rapid pace, allowing for broader eligibility for transcatheter intervention for severe TR. Many of the current TTVI devices appear to have safe, efficacious profiles and can mostly accommodate the various TR etiologies. The current randomized clinical trials should help answer the unknowns related to patient selection, device efficacy and long-term outcomes. As clinical experience continues to grow and newer-generation devices are developed, TTVI appears likely to follow the path of transcatheter aortic and mitral interventions as an accessible and beneficial therapeutic option for this underserved patient population.

## Disclosures

RH reports speaker fees from Abbott Structural, Baylis Medical, Edwards Lifesciences, and Philips Healthcare; she has institutional consulting contracts for which she receives no direct compensation with Abbott Structural, Boston Scientific, Edwards Lifesciences, Medtronic, and Novartis; she has Equity with NaviGate. The remaining authors have no disclosures to report.

## Author contributions

DB wrote the initial draft. RH performed extensive editing and content review. All authors contributed to the article and approved the submitted version.

## Conflict of interest

The authors declare that the research was conducted in the absence of any commercial or financial relationships that could be construed as a potential conflict of interest.

## Publisher’s note

All claims expressed in this article are solely those of the authors and do not necessarily represent those of their affiliated organizations, or those of the publisher, the editors and the reviewers. Any product that may be evaluated in this article, or claim that may be made by its manufacturer, is not guaranteed or endorsed by the publisher.

## Abbreviations

3D: three dimensional
6MWD: 6-min walk distance
CCT: cardiac computed tomography
CIED: cardiac implantable electronic device
CMR: cardiac magnetic resonance imaging
KCCQ: Kansas City Cardiomyopathy Questionnaire
LV: Left ventricle
NYHA: New York Heart Association
PA: Pulmonary artery
PHT: Pulmonary hypertension
RA: Right atrial
RV: Right ventricular
TAPSE: Tricuspid annular plane systolic excursion
TEE: Transesophageal echocardiography
TEER: Transcatheter edge-to-edge repair
TTE: Transthoracic echocardiography
TR: Tricuspid regurgitation
TTVI: Transcatheter tricuspid valve intervention
TV: Tricuspid valve
